# A New Method for the Isolation of Ergosterol and Peroxyergosterol as Active Compounds of *Hygrophoropsis aurantiaca* and in Vitro Antiproliferative Activity of Isolated Ergosterol Peroxide

**DOI:** 10.3390/molecules21070946

**Published:** 2016-07-21

**Authors:** Renata Nowak, Marta Drozd, Ewaryst Mendyk, Marta Lemieszek, Olga Krakowiak, Wanda Kisiel, Wojciech Rzeski, Katarzyna Szewczyk

**Affiliations:** 1 Department of Pharmaceutical Botany, Medical University of Lublin, 1 Chodźki Str., 20-093 Lublin, Poland; medea_ua@tlen.pl (M.D.); ola.krakowiak@wp.pl (O.K.); k.szewczyk@umlub.pl (K.S.); 2 Analytical Laboratory, Faculty of Chemistry, University of Maria Curie-Sklodowska, 3/27 M.C. Skłodowska Sq., 20-031 Lublin, Poland; emendyk@umcs.pl; 3 Department of Medical Biology, Institute of Rural Health, 2 Jaczewskiego Str., 20-090 Lublin, Poland; martalemieszek@gmail.com (M.L.); rzeski.wojciech@imw.lublin.pl (W.R.); 4 Department of Phytochemistry, Institute of Pharmacology, Polish Academy of Sciences, 12 Smętna Str., 31-343 Kraków, Poland; kisielw@if-pan.krakow.pl; 5 Department of Virology and Immunology, Institute of Microbiology and Biotechnology, University of Maria Curie-Sklodowska, 19 Akademicka Str., 20-033 Lublin, Poland

**Keywords:** TLC-DPPH, *Hygrophoropsis aurantiaca*, ergosterol, peroxyergosterol, antiproliferative effect, cytotoxicity

## Abstract

In the present study, ergosterol peroxide and ergosterol were isolated for the first time from fresh fruit bodies of *Hygrophoropsis aurantiaca* (False Chanterelle). The substances were characterized mainly by spectroscopic methods (^1^H-NMR, ^13^C-NMR, DEPT-45, DEPT-90, DEPT-135, 2D-NMR). In our study, a new specific thin layer chromatographic method was developed for determination of ergosterol and ergosterol peroxide in *H.* *aurantiaca* extract. The method is based on the separation of *n*-hexane extract on silica gel (Silica Gel G) TLC plates using the optimized solvent system toluene/ethyl acetate (3:1; *v*/*v*). The main advantages of the developed method are the simplicity of operation and the low cost. The in vitro study results revealed the antiproliferative properties of ergosterol peroxide against LS180 human colon cancer cells. The described effect was attributed both to altered mitochondrial activity and decreased DNA synthesis. Additionally, in the same concentration range the investigated compound was not toxic to CCD 841 CoTr human colon epithelial cells. The present study suggests that fruit bodies of *H. aurantiaca* have great potential for producing substances and extracts with potential applications in medicine.

## 1. Introduction

Wild mushrooms have recently gained much attention as important sources of chemically interesting and biologically active secondary metabolites for the development of new pharmaceutical agents. Mushrooms have been reported to have hypotensive, anticholesterolemic, hypoglycemic, antiinflammatory, antibacterial, antiviral, and antifungal properties [1,2,3]. Furthermore, their role in prevention and treatment of various cancers (breast, stomach, liver, colorectal, lung, cervical and endometrium) has been reported [4,5]. The pharmacological properties of mushrooms are associated with the presence of chemical compounds such as phenolics, terpenes, steroids, polysaccharides, and proteins [6,7].

*Hygrophoropsis aurantiaca* (Wulf.: Fr.) J. Schröt belongs to the Hygrophoropsidaceae family (Kingdom: Fungi, Phylum = Division: Basidiomycota, Class: Basidiomycetes, Subclass: Agaricomycetidae, Order: Boletales, Genus: *Hygrophoropsis* (J. Schröt.) Maire) [8]. *H. aurantiaca* is an orange funnel-shaped mushroom commonly known as the False Chanterelle, distributed in Europe and North America in summer and autumn. It is listed as edible by some authors, but poisonous by others. There is a little information about chemical constituents of *H. aurantiaca.* Only some substances of nutritional value were analyzed [9,10]. Heleno et al. [10] analyzed tocopherols of Portuguese wild mushrooms and they found that *H. aurantiaca* presented the highest phenols and tocopherols contents, 7.90 ± 0.29 mg GAE/g and 1.94 ± 0.10 µg/g, respectively. Among tocopherols the authors identified α-, β-, γ- and δ- tocopherol. Furthermore, the phenols contents was in agreement with the antioxidant properties of this species [10,11].

Ergosterol is frequently found in extracts from fungi, because it is a part of their cytoplasmic membrane. Ergosterol is the provitamin D2 and recently vitamin D2 was shown to contribute to the prevention of prostate and colon cancer [12]. Ergosterol peroxide (EP) seems to be a wildly distributed among fungi natural compounds, too. It has been obtained from some species of fungi and marine organisms [13,14,15]. In some recent studies peroxyergosterol showed potent antioxidant and antiinflammatory activities and inhibitory effects on some cancer cell lines [16,17,18,19,20]. On the other hand, Gao et al. [21] reported a selective inhibitory activity against *Crotalus adamenteus* venom phospholipase A_2_ enzyme for ergosterol peroxide isolated from the fungus *Lactarius hatsudake*. Furthermore, Zhu et al. [15] showed that EP from *Cordyceps cicadae* is able to suppress TGF-β 1–induced fibroblasts activation in NRK-49F (a rat kidney fibroblast cell line). This compound possess antibacterial activity, too [22].

**Figure 1:**
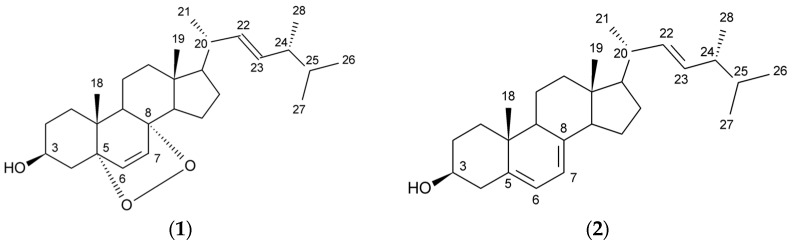
Chemical structures of the isolated compounds: ergosterol peroxide (**1**); ergosterol (**2**).

In this work ergosterol and ergosterol peroxide (5α,8α-epidioxy-22*E*-ergosta-6,22-dien-3β-ol) (Figure 1) were isolated from ethanolic extracts of *H. aurantiaca*. Their structures were established using spectroscopic methods. This is the first report that deals with the occurrence of these compounds in the investigated mushroom species. The method that allowed the isolation of these compounds is new. In addition, the antiproliferative activity of isolated ergosterol peroxide against LS180 human colon cancer cells was determined.

## 2. Results and Discussion

Both isolated compounds were identified on the basis of their UV, EI-MS, 1D- and 2D-NMR data. The melting point of compound **1** was between 179–182 °C. The ^13^C-NMR spectrum (Figure S1) showed the presence of two disubstituted olefins (δ 130.8 (C-7), 132.3 (C-23), 135.2 (C-6), 135.4 (C-22)), indicating that the sterol fragment of compound **1** is an ergosterol derivative. Besides, two oxygenated quaternary carbons of δ 79.4 (C-5) and 82.2 (C-8) suggested the presence of a peroxide structure. The signals at δ 6.25 and δ 6.52 (d, *J* = 8 Hz, 2H, H-6, H-7) in the ^1^H-NMR spectrum revealed the presence of a disubstituted double bond which were correlated with carbon signals of δ 135.4 (C-6) and δ 130.8 (C-7) in HMBC spectrum. The ^1^H-NMR spectrum (Figure S2) showed also signals for six methyl groups, two singlets at δ 0.83 and 0.89, and four doublets at δ 0.82 (*J* = 6.8 Hz), 0.83 (*J* = 6.6 Hz), 0.91 (*J* = 6.8 Hz) and 1.00 (*J* = 6.7 Hz). Moreover, a multiplet at δ 3.98, characteristic of a steroid oxymethine signal located at C-3, was observed. The 2D-NMR (Figures S3 and S4) experiments confirmed that compound **1** is a steroid, containing a peroxy function at C-5/C-8 and two double bonds in the side chain and at C-6/C-7. To determine the bond order of individual atoms DEPT-45, DEPT-90 and DEPT-135 spectra (Figures S5–S7) were additionally performed to compound **1**. In the EI-MS (70eV) spectrum the ions of *m*/*z* 428 [M^+^], 396 [M^+^ − O_2_], 363 [M^+^ − (O_2_ + CH_3_ + H_2_O)] and 337 [M^+^ − (O_2_ + C_3_H_5_ + H_2_O)] were assigned. Spectral analyses from NMR (Table 1) and mass spectroscopy suggested that the obtained compound was ergosterol peroxide (5,8-epidioxy-5α,8α-ergosta-6,22*E*-dien-3β-ol). The ^1^H- and ^13^C-NMR data were in good agreement with the literature values [13,14,19,23]. In this paper ergosterol peroxide was isolated from *H.* *aurantiaca* first time, so it is a new compound for this species.

Compound **2** was identified based on mass spectra in the negative mode and by NMR spectroscopy as ergosterol (ergosta-5,7,22-trien-3β-ol; mp uncorr. 167–169 °C) [19,24]. The difference in molecular formula between compound **2** and **1** was two oxygen atoms, implying that compound **1** is a peroxidated derivative of ergosterol (**2**).

The method mainly used for the analysis of sterolic fractions involves several steps, namely, the saponification of the oil to remove triglycerides, the fractionation of the unsaponifiable matter into several classes of compounds by thin layer chromatography, and their subsequent analysis by gas chromatography as trimethylsilyl derivatives on non-polar capillary gas chromatography columns. A number of various methods have been reported for the isolation and identification of sterols in fungi and higher plants. In most cases the extracts were first subjected to alkaline saponification before chromatographic analysis [24,25]. The saponification is most often accomplished by means KOH or NaOH in water or water-ethanolic media. The most commonly reported in the literature and efficient methods of extraction for sterols are supercritical fluid extraction [26,27], microwave-assisted extraction [28] and ultrasonic extraction using organic solvents such as *n*-hexane, dichloromethane or methanol [29].

For determining ergosterol and its derivatives content in mushrooms gas chromatography coupled to mass spectrometry and reversed-phase liquid chromatography appears to be an advantageous techniques which are the methods capable of separating free ergosterol from the ergosteryl derivatives [30,31].

Thin layer chromatography (TLC) is one of the techniques using to the identification and separation of sterols. Krzyczkowski et al. [13] have developed a new densitometric method for the quantitative determination of ergosterol peroxide in hexane extracts of various edible mushroom species. For sterols separation the most commonly solvents for TLC on silica gel are these containing different proportions of chloroform-methanol-water [32].

In our study, a new specific thin layer chromatographic method was developed for determination of ergosterol and ergosterol peroxide in *H.* *aurantiaca* extract. The method is based on the separation of *n*-hexane extract on silica gel (Silica Gel G) TLC plates using the optimized solvent system toluen/ethyl acetate (3:1; *v*/*v*).

Both isolated compounds belong to the group of sterols and have a lipophilic character. It is surprising that they were isolated from the ethanol extract, typical of polyphenolic compounds with differential structure and generally hydrophilic character. This is only an apparent contradiction arising from the phenomenon of co-solution of compounds in rich, multi-component mixtures of natural products.

**Figure 2:**
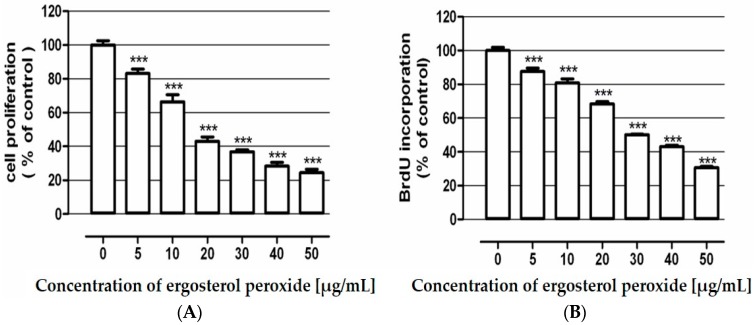
Antiproliferative effect of ergosterol peroxide in human colon adenocarcinoma cell line LS180. Cells were exposed to culture medium alone (control) and the medium supplemented with tested compound in concentrations ranging from 5–50 µg/mL for 96 h (**A**) and 48 h (**B**). Cell proliferation was quantified by MTT test (**A**) or BrdU assay (**B**). Results are presented as the mean ± SEM of 5–6 measurements. *** *p* < 0.001 vs. control, one-way ANOVA test; *post* test: Tukey.

The antiproliferative activity of ergosterol peroxide in the LS180 human adenocarcinoma cell line was assessed. In the first set of experiments, investigated compound at the concentration range (5–50 μg/mL) was applied and a 3-(4,5-dimethylthiazol-2-yl)-2,5-diphenyltetrazolium bromide (MTT) test was performed after 96 h of cancer cell treatment. The obtained results revealed significant inhibition of colon cancer cells proliferation after incubation with ergosterol peroxide (Figure 2A). The observed effect was dose dependent, with an IC_50_ value of 17.3 µg/mL. At the highest concentration (50 μg/mL) the tested compound reduced LS180 cell proliferation down to 75.48% versus control conditions.

In order to reveal the influence of ergosterol peroxide on DNA synthesis within the cancer cells, the investigated cell line LS180 was exposed for 48 h to increasing concentrations (5–50 μg/mL) of tested compound followed by incubation with BrdU. Incorporation of this thymidine analogue was significantly affected in colon cancer cells treated with ergosterol peroxide (Figure 2B). The investigated compound decreased cancer cell proliferation in the whole applied dose range reducing the cell division by up to 69.25 µg/mL (IC_50_ = 32.3 µg/mL).

**Figure 3:**
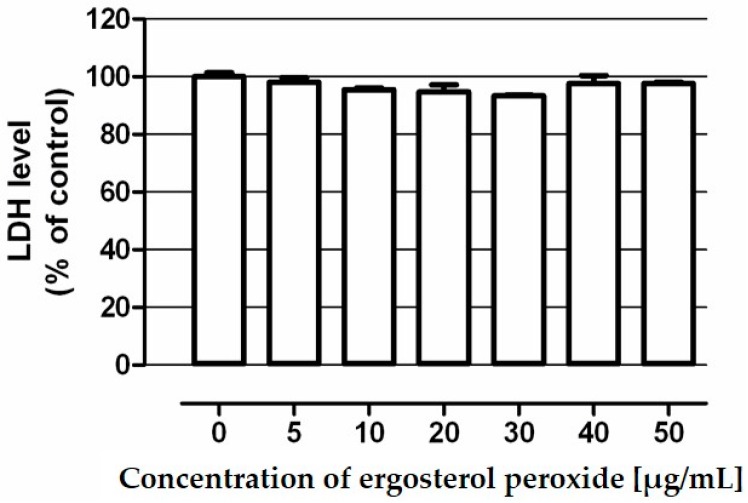
Cytotoxicity of ergosterol peroxide in human colon epithelial cell line CCD 841 CoTr. Cells were exposed to culture medium alone (control) and the medium supplemented with tested compound in concentrations ranging from 5–50 µg/mL for 48 h. Compound cytotoxicity was measured by means of the LDH assay. Results are presented as the mean ± SEM of 4 measurements.

To further investigate the cytotoxicity of ergosterol peroxide against human colon epithelial cells several concentrations of tested compound were applied (5–50 μg/mL) for 48 h in CCD 841 CoTr cells. Cell viability was determined by means of a lactate dehydrogenase (LDH) test. Performed studies revealed that the tested agent was nontoxic to colon epithelial cells in the whole range of investigated concentrations (Figure 3).

The finding of these constituents in *H. auriantiaca* suggests that this mushroom may constitute a source of new pharmacological active compounds. In vitro studies revealed antiproliferative abilities of ergosterol peroxide against human colon cancer cells LS180. The observed decrease of cell proliferation was directly proportional to the used concentration. The described effect was attributed both to altered mitochondrial activity and decreased DNA synthesis. Concomitantly the investigated compound was not toxic in the same concentration range to human colon epithelial cells CCD 841 CoTr.

## 3. Experimental Section

### 3.1. Reagents and Materials

All solvents used for the study (pure or high purity) were purchased from Polish Reagents (POCH, Gliwice, Poland).Ergosterol standard and 2,2-diphenyl-1-picrylhydrazyl (DPPH) were purchased from Sigma-Aldrich Fine Chemicals (St. Louis, MO, USA).

Human colon epithelial cell line CCD 841 CoTr was purchased from the ATCC (American Type Culture Collection, Menassas, VA, USA). Human colon adenocarcinoma cell line LS180 was obtained from ECACC (European Collection of Cell Cultures, Centre for Applied Microbiology and Research, Salisbury, UK). CCD 841 CoTr cells were grown in Dulbecco’s Modified Eagle Medium (DMEM; Sigma-Aldrich).

Fresh fruit bodies (650 g) of *H.* *aurantiaca* were collected in August 2014 in the village of Luta (51°31′1.33″ N; 23°22′12.60″ E) near Włodawa (Poland). Multi-stage extraction was used. After macerating twice with ethanol (room temperature, 7 days) fruit bodies were subjected to exhaustively extraction using ultrasonic bath (40 °C, 2 × 45 min). Crude ethanol extracts were brought to dryness in vacuo and redissolved in distilled water. After partitioning with hexane, EtOAc and butanol, the combined layers were concentrated and freeze dried to give the hexane (H), ethyl acetate (EA), and butanol (B) fractions. The obtained fractions were tested for the presence of terpenes and polyphenols with antioxidant activity using a TLC-DPPH method.

### 3.2. Apparatus

^1^H-NMR, ^13^C-NMR, Distortionless Enhancement by Polarization Transfer (DEPT-45, DEPT-90, DEPT-135; 500 MHz) and 2D-NMR ((Correlation Spectroscopy (^1^H,^1^H COSY), Heteronuclear Multiple Quantum Coherence (^1^H,^13^C HMQC) and Heteronuclear Multiple Bond Correlation (HMBC)) were recorded on an Avance III instrument (Bruker, Fallanden, Switzerland) using solutions in deuterochloroform (CDCl_3_) with tetramethylsilane (Si(CH_3_)_4_) as an internal standard. Depending on the spectrum performed different probes were used (microcapillary TXI probe for ^1^H; the BBO-probe for ^13^C). Purity and identity (mass spectra) of the isolated compounds were determined using HPLC-MS/MS (Agilent 1200 HPLC system, Agilent Technologies, Waldronn, Germany) coupled with a QTRAP 4000 mass detector (ABSciex, Concord, ON, Canada). The melting points were measured using a Melt-Temp apparatus (Bibby Scientific Limited, Staffordshire, UK) with open capillary tubes and values were uncorrected.

### 3.3. Preparative Thin-Layer Chromatography

In the course of research conducted in the Department of Pharmaceutical Botany in Lublin, which was focused on the antioxidant activity of ethanol extracts of macrofungi growing in Poland, *H.* *aurantiaca* was identified as one of the most active species. Consequently, we tried to identify and isolate the compounds responsible for this activity.

**Figure 4:**
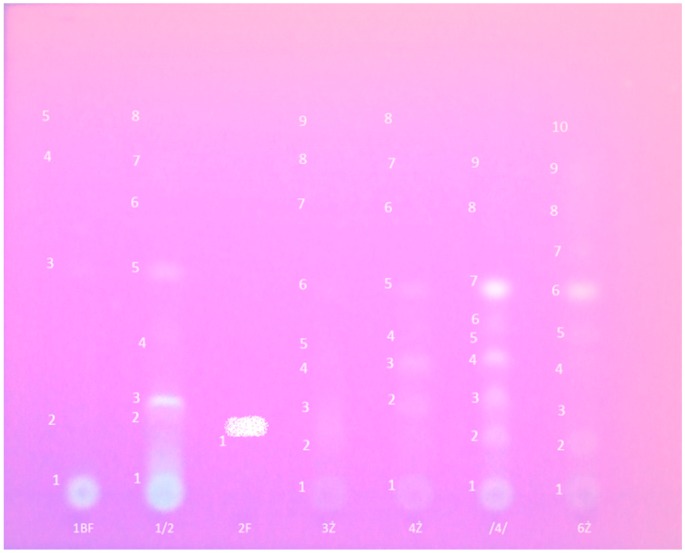
The chromatogram of the 1–6 fractions after preparative thin layer chromatography.

All three crude extracts (H, EA, and B) were tested for the presence of terpenes and polyphenols using the TLC-DPPH method [33]. For preliminary chromatographic analysis of all extracts 1D- and 2D-TLC on different stationary and various eluents were used. Based on the results of the initial TLC, the hexane (H; 208 mg) fraction was selected for preparative separation (Silica Gel G; a mixture of toluene/ethyl acetate (3:1, *v*/*v*) as eluant). Depending on the degree of reduction (discoloration) after spraying with reagent W1 (0.1% ethanolic solution of DPPH) bands for preparation were determined. Additionally, part of the cut plate was immersed in the reagent W1. The separated bands after scraping from the plates were extracted twice with ethanol using a laboratory shaker for 5 h. The third time, ultrasound-assisted extraction (1 h) was used. The combined eluates were centrifuged, filtered, and evaporated to dryness. Eight fractions were obtained, which were re-analyzed using a TLC-DPPH method. The results of preparative TLC of the hexane layer are presented in Table 2 and Figure 4.

### 3.4. Preparative Column Chromatography

To isolate active compound fraction 2 (one intensive spot with high antioxidant activity and slight contamination) was selected. It was fractionated by silica gel column chromatography (Silica gel; 230 mesh; Machery Nagel; Merck; Darmstadt, Germany) using a step-wise gradient of hexane and chloroform (hexane; hexane/chloroform 9:1 to 7:3; hexane/chloroform 7:3 with 5% or 10% ethyl acetate). Nineteen fractions of varying composition and activity as compared to DPPH were obtained. Compound **1** (white crystalline needles; 46 mg) was crystallized from fractions 10–13 (eluted with hexane/chloroform, 7:3, *v/v* + 5% ethyl acetate; re-crystallization from methanol). In the remaining solution another precipitate was formed after a few days. After recrystallization from methanol compound **2** (white lamellas; 12 mg) was isolated.

### 3.5. Examination of Ergosterol Peroxide Antiproliferative Activity

Ergosterol peroxide influence on cancer cells proliferation was determined by a MTT metabolism assay as well as a BrdU incorporation immunoassay. The BrdU test was conducted using Cell Proliferation ELISA BrdU according the manufacturer’s instructions (Roche Diagnostics GmbH, Penzberg, Germany). Both studies were performed in human adenocarcinoma cell line LS180. Details of the procedures were described elsewhere [34].

### 3.6. Assessment of Ergosterol Peroxide Cytotoxicity

Ergosterol peroxide cytotoxicity was examined by a colorimetric test based on the measurement of lactate dehydrogenase (LDH) release. Studies were performed in human colon epithelial cell line CCD 841 CoTr using In Vitro Toxicology Assay Kit Lactate Dehydrogenase Based (Sigma). Details of the procedure was described elsewhere [34].

### 3.7. Statistical Analysis

The data were presented as the mean value and standard error of the mean (SEM). Statistical analysis was performed using the one way-ANOVA with the Tukey post-hoc test and column statistics used for comparisons. Significance was accepted at *p* < 0.05. The IC_50_ value (concentration causing proliferation inhibition by 50% compared to control) was calculated according to the Litchfield and Wilcoxon method [35].

## 4. Conclusions

The present study has been focused on the isolation of two lipophilic compounds from fresh fruit bodies of *H. aurantiaca*. Thin layer chromatography is one of the techniques used in the identification and separation of sterols. For determination of ergosterol and ergosterol peroxide a new specific thin layer chromatographic method was developed. The method is based on the separation of *n*-hexane extract on TLC silica gel plates using the optimized solvent system toluene/ethyl acetate (3:1; *v*/*v*). An important advantage of thin-layer chromatography is the simplicity of the analysis, without need of use of expensive equipment. The optimized TLC method allowed us to obtain two pure compounds with interesting biological activities. The finding of ergosterol and ergosterol peroxide in *H. auriantiaca* suggests that this mushroom may constitute the source of new pharmacologically active compounds.

## Figures and Tables

**Table 1:** ^1^H- and ^13^C-NMR data, and ^1^H–^13^C HMBC correlation for ergosterol peroxide (**1**, 500 MHz, CDCl_3_, δ in ppm, *J* in Hz).

	^1^H	^13^C	HMBC (^1^H → ^13^C)
1	1.73, dd, *J* = 13.8, 3.4	34.7	C-2, C-3, C-5, C-6, C-10, C-19
2	-	30.1	
3	3.98, m	66.5	
4	-	37.0	
5	-	82.2	
6	6.25, d, *J* = 8.5	135.4	C-5, C-8, C-10
7	6.52, d, *J* = 8.6	130.8	C-5, C-8, C-9, C-14
8	-	79.4	
9	-	51.1	C-7, C-8
10	-	36.9	
11	1.23, m; 1.55, m	20.6	
12	1.27, m; 1.98, m	39.4	
13	-	44.6	
14	1.59, m	51.7	C-6, C-7, C-8, C-9, C-13
15	1.42, m; 1.66, m	23.4	
16	1.33, m; 1.81, m	28.7	C-13, C-14, C-18; C-18
17	1.25, m	56.2	C-12, C-16
18	0.83, s	12.9	C-13, C-14, C-17
19	0.89, s	18.2	C-1, C-5, C-9, C-10
20	2.05, m	39.7	
21	1.00, d, *J* = 6.7	20.9	C-17, C-20, C-22
22	5.16, dd, *J* = 7.5, 15.3	135.2	C-17, C-21, C-23, C-24
23	5.14, dd, *J* = 8.0, 15.3	132.3	C-20, C-22, C-24, C-28
24	1.86, m	42.8	C-22, C-23, C-25, C-26, C-27, C-28
25	1.6, m	33.1	C-24, C-26, C-27
26	0.82, d, *J* = 6.8	19.6	C-24, C-25, C-27
27	0.83, d, *J* = 6.6	20.0	C-24, C-25, C-26
28	0.91, d, *J* = 6.8	17.6	C-24, C-24, C-25

**Table 2:** TLC bands of the hexane layer during preparative thin-layer chromatography (Fertigplatten Gel (Merck, Darmstadt, Germany), toluene/ethyl acetate (3:1, *v*/*v*), reagent W1).

TLC Band	The Color under UV Light at 365 nm/degree of Discoloration W1	Fraction	Rf Range
1	White-purple/discoloration	1	0
2	No color/no discoloration	1/2	0.1–0.25
3	Purple/strong discoloration	2	0.25–0.3
4	Yellow/cloudy discoloration	2/3	0.35–0.45
5	Yellow/cloudy discoloration	3	0.5–0.6
6	No color/discoloration	4	0.6–0.75
7	Blue/cloudy discoloration	5	0.76–0.8
8	No color/no discoloration	6	0.8–0.9

## Supplementary Materials

The following are available online at: http://www.mdpi.com/1420-3049/21/7/946/s1, scans of ^13^C-NMR, ^1^H-NMR, 2D-NMR, DEPT-45, DEPT-90, DEPT-135 of compound **1**.
