# Supplementary material for: A New Method for the Isolation of Ergosterol and Peroxyergosterol as Active Compounds of Hygrophoropsis aurantiaca and in Vitro Antiproliferative Activity of Isolated Ergosterol Peroxide
Source: Molecules. 2016 Jul 21;21(7):946. doi: 10.3390/molecules21070946 (PMC6273287; doi:10.3390/molecules21070946)
Supplement: Supplementary file 1 [file molecules-21-00946-s001.pdf]

Supplementary Materials: A New Method for the Isolation of Ergosterol and Peroxyergosterol as an Active Compounds of *Hygrophoropsis aurantiaca* and in Vitro Antiproliferative Activity of Isolated Ergosterol Peroxide

Renata Nowak, Marta Drozd, Ewaryst Mendyk, Marta Lemieszek, Olga Krakowiak, Wanda Kisiel, Wojciech Rzeski and Katarzyna Szewczyk

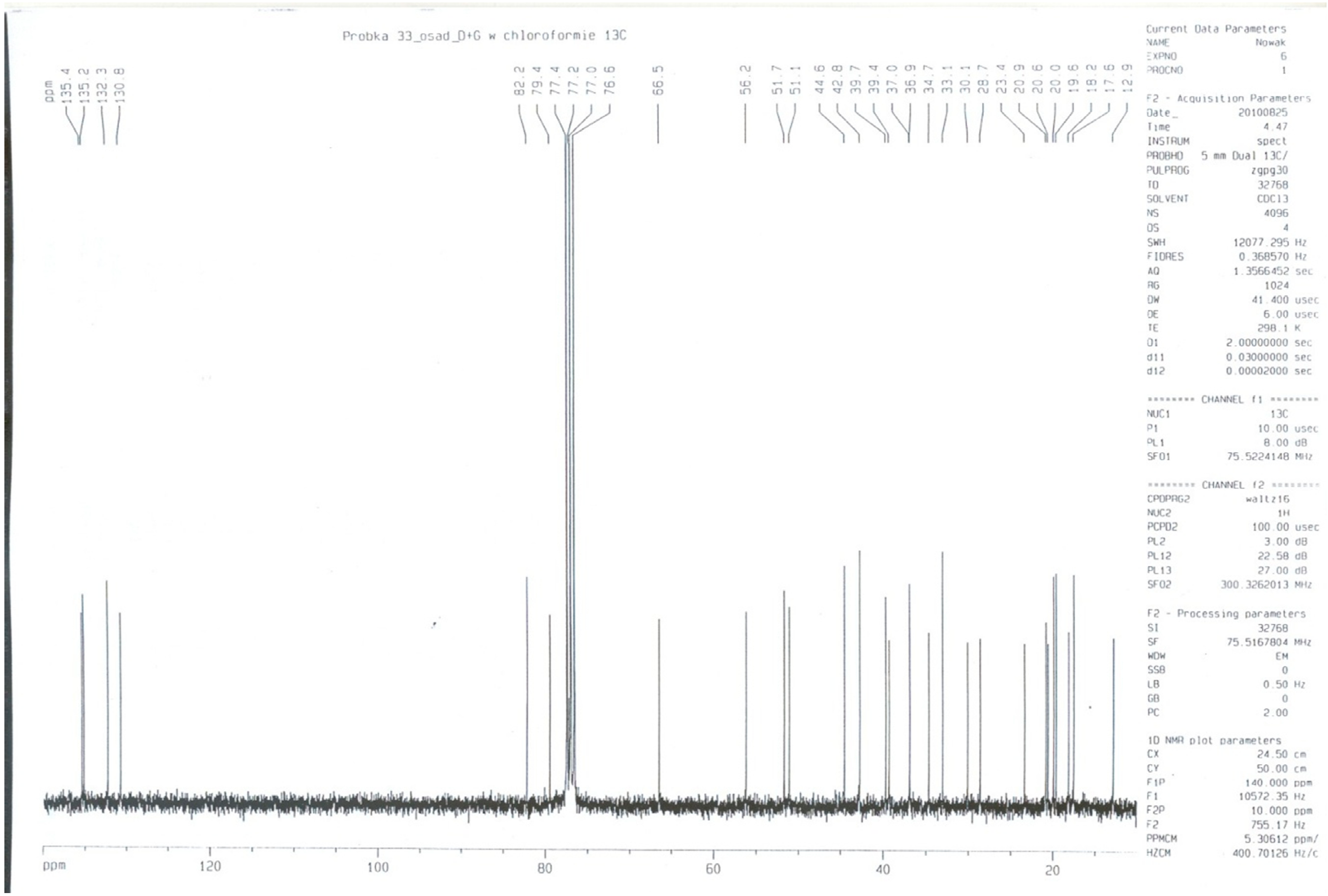

Figure S1. ^{13}C NMR spectrum of ergosterol peroxide.

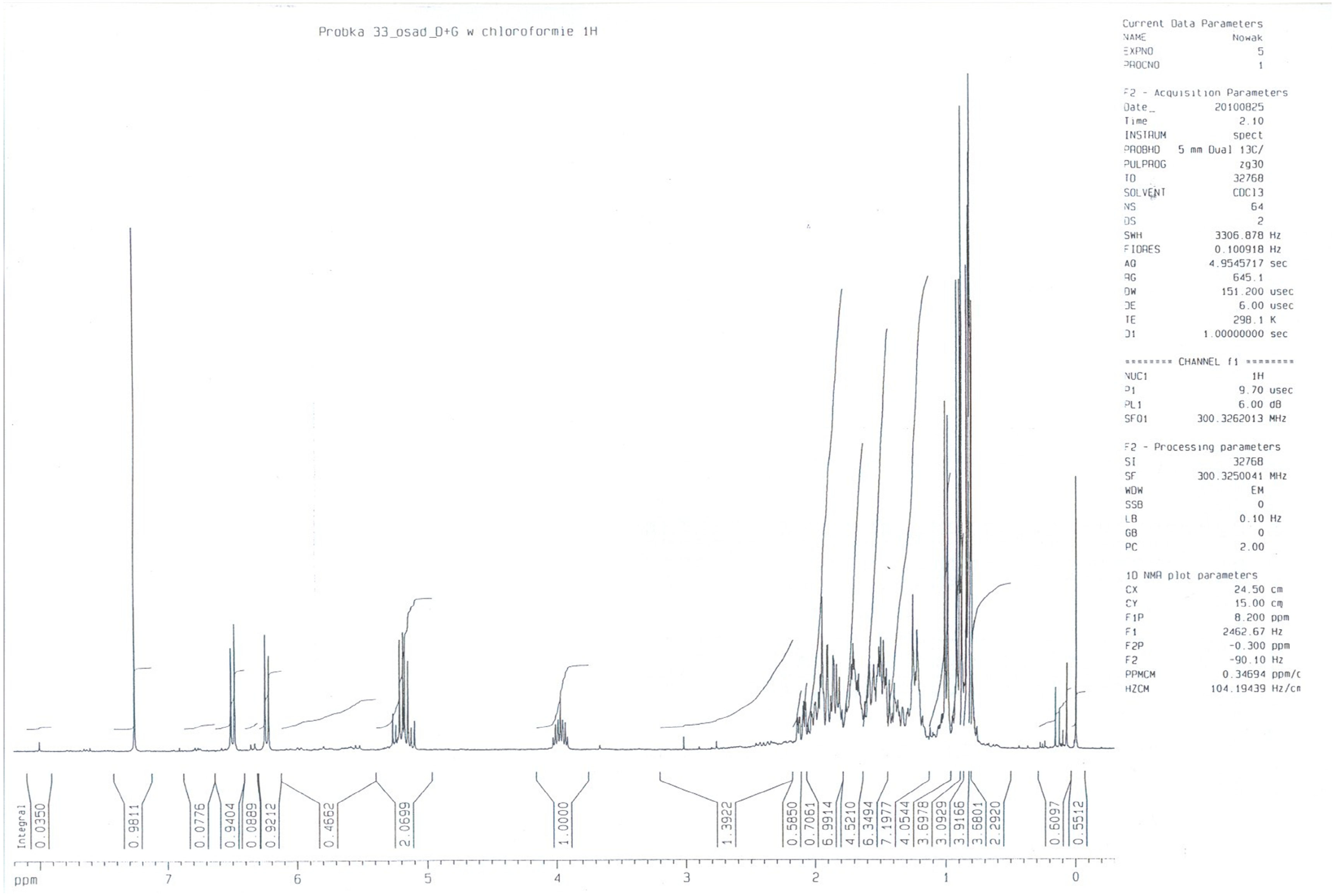

Figure S2. ¹H NMR spectrum of ergosterol peroxide.

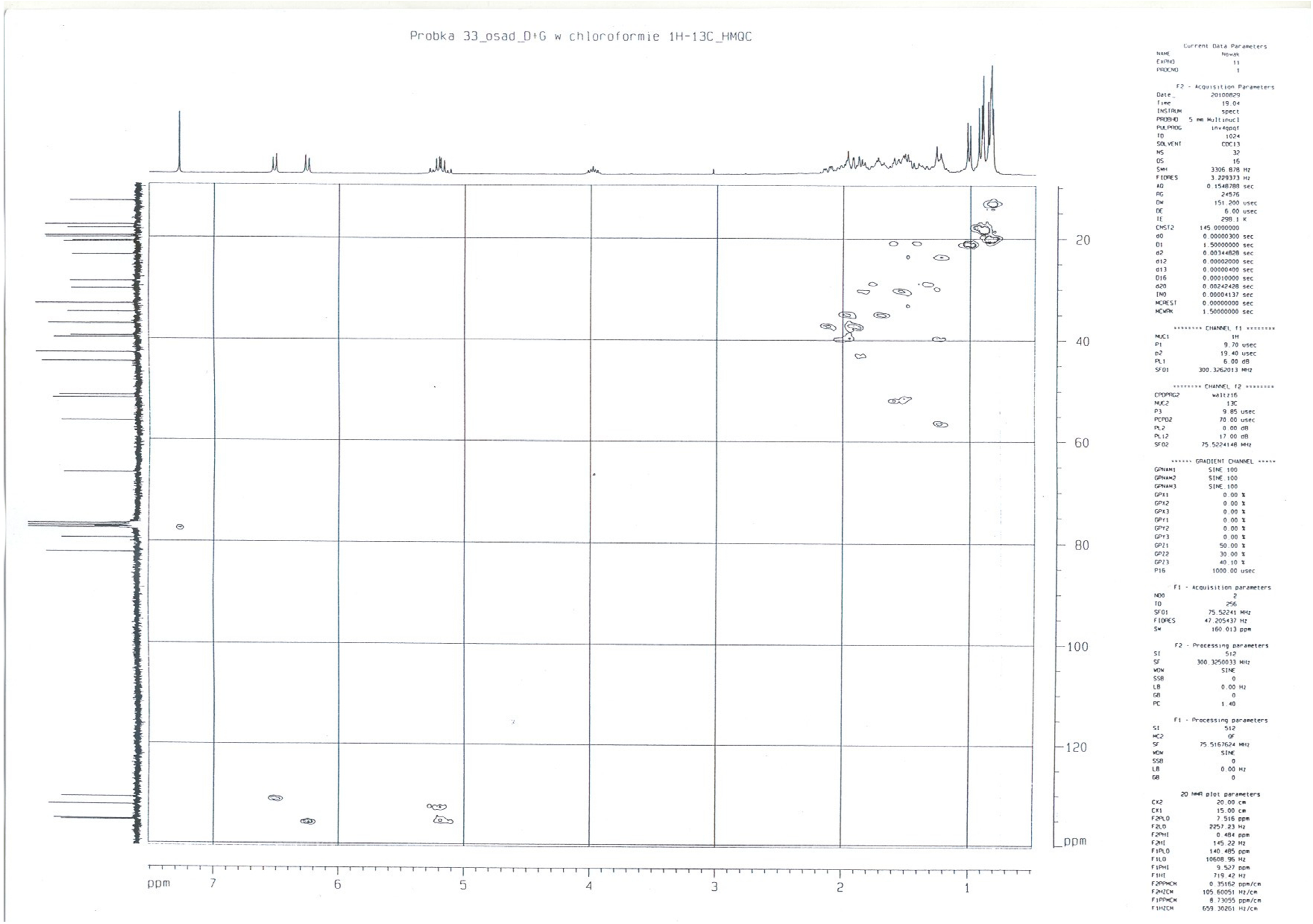

Figure S3. ^{1}H–^{13}C HMQC spectrum of ergosterol peroxide.

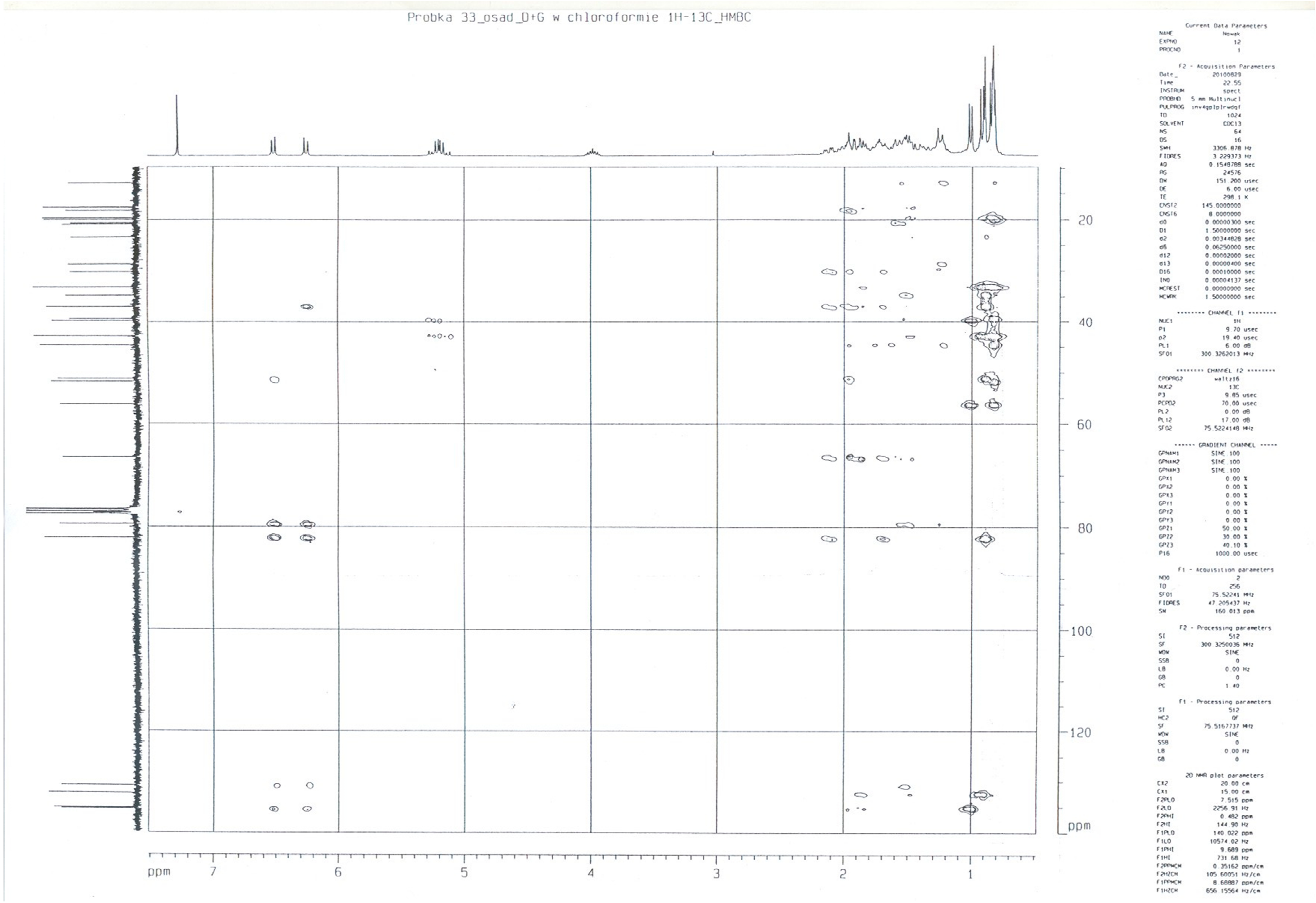

Figure S4. ^{1}H ^{13}C HMBC spectrum of ergosterol peroxide.

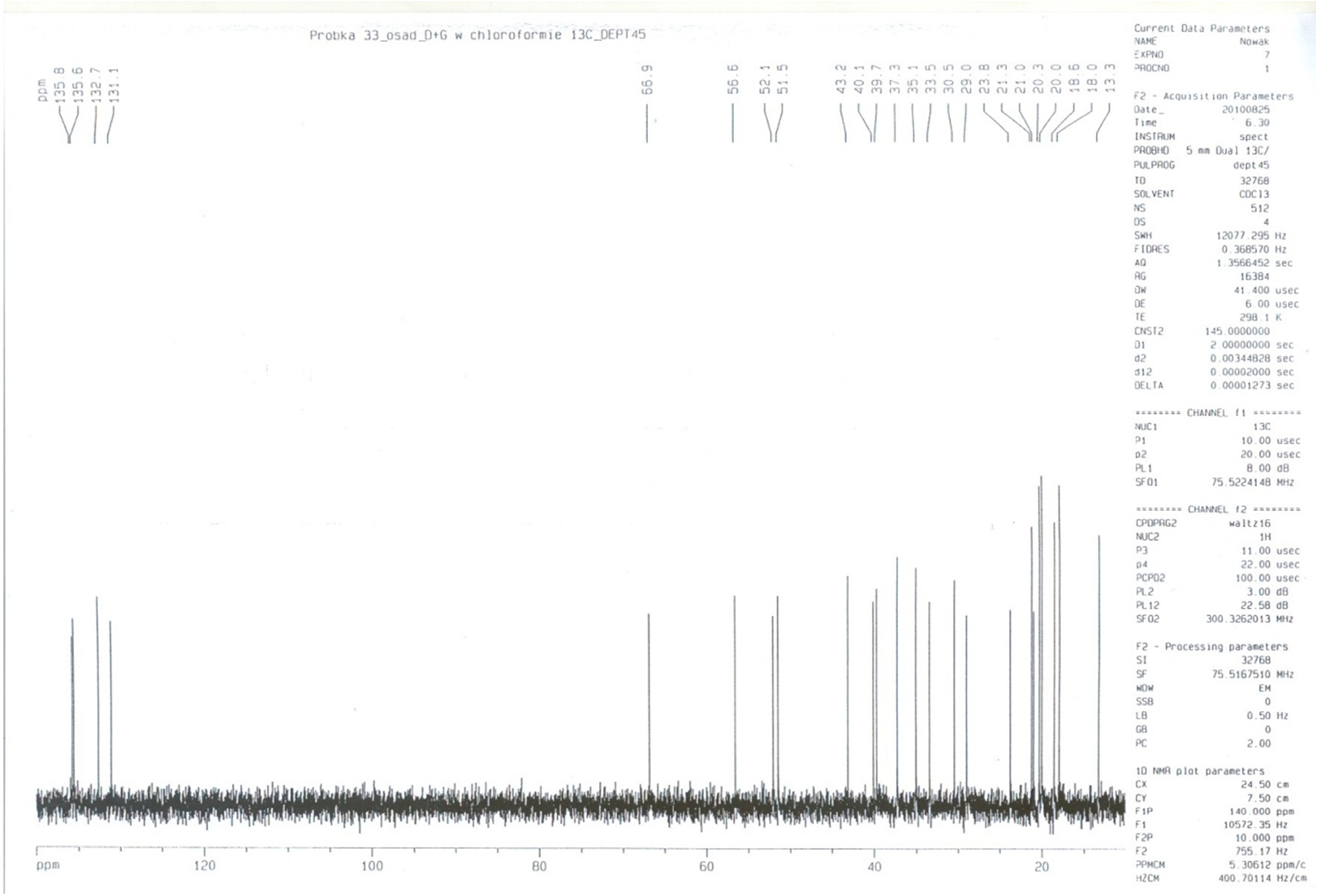

Figure S5. ^{13}C DEPT-45 spectrum of ergosterol peroxide.

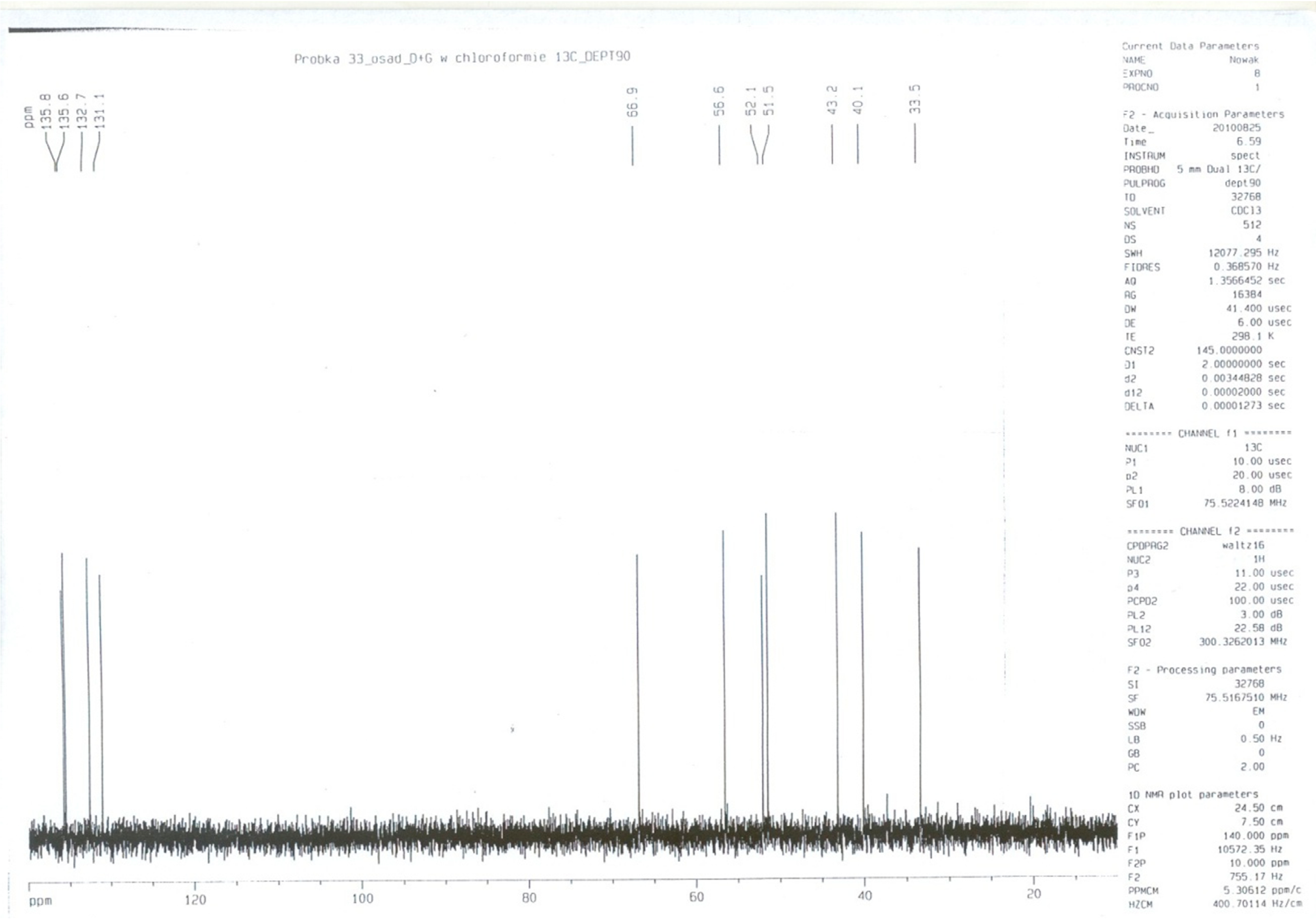

Figure S6. ^{13}C DEPT-90 spectrum of ergosterol peroxide.

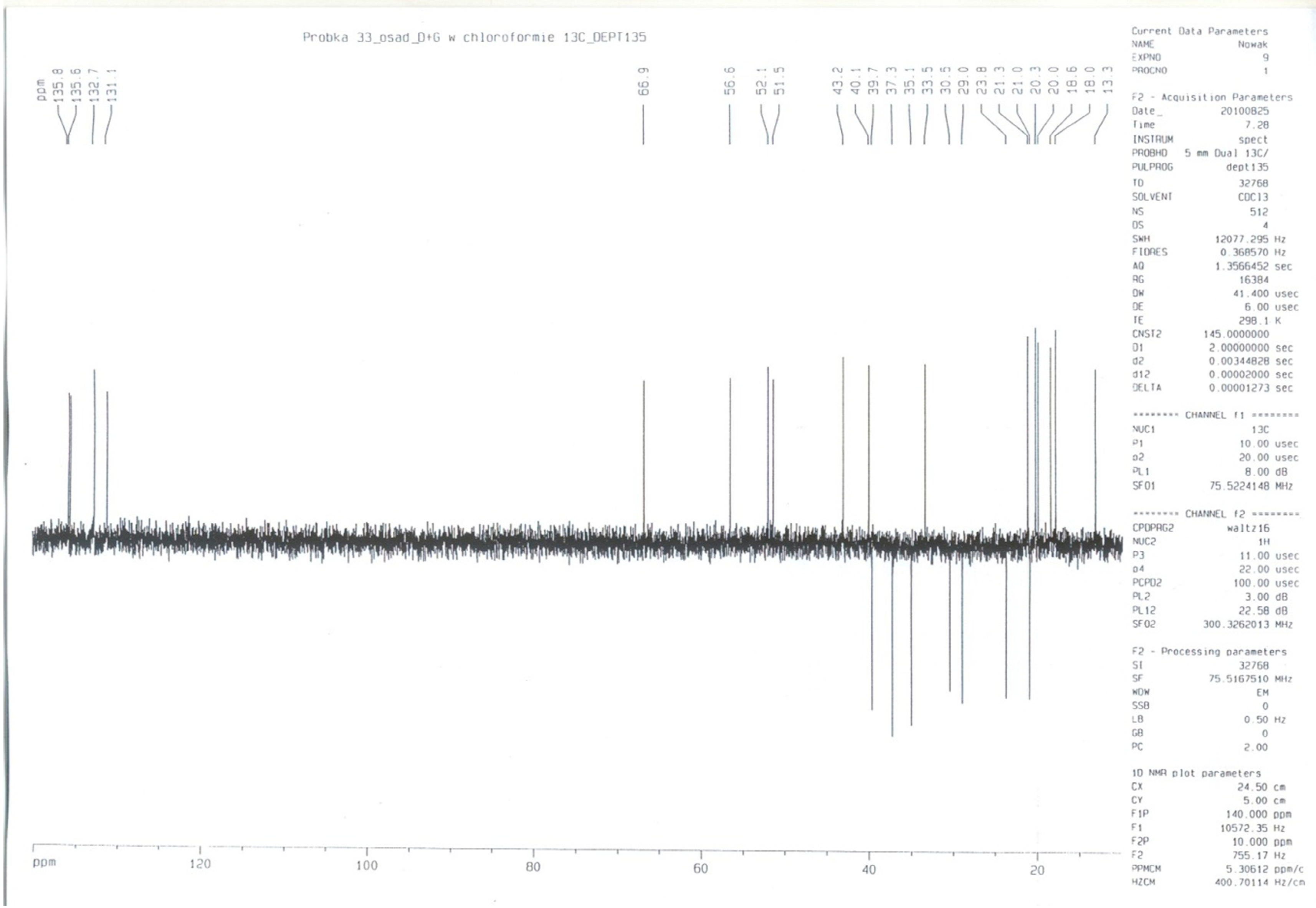

Figure S7. ^{13}C DEPT-135 spectrum of ergosterol peroxide.
